# Retroviral Integration Mutagenesis in Mice and Comparative Analysis in Human AML Identify Reduced *PTP4A3* Expression as a Prognostic Indicator

**DOI:** 10.1371/journal.pone.0026537

**Published:** 2011-10-20

**Authors:** Renée Beekman, Marijke Valkhof, Stefan J. Erkeland, Erdogan Taskesen, Veronika Rockova, Justine K. Peeters, Peter J. M. Valk, Bob Löwenberg, Ivo P. Touw

**Affiliations:** 1 Department of Hematology, Erasmus University Medical Center, Rotterdam, The Netherlands; 2 Department of Biostatistics, Erasmus University Medical Center, Rotterdam, The Netherlands; Kyushu Institute of Technology, Japan

## Abstract

Acute myeloid leukemia (AML) results from multiple genetic and epigenetic aberrations, many of which remain unidentified. Frequent loss of large chromosomal regions marks haplo-insufficiency as one of the major mechanisms contributing to leukemogenesis. However, which haplo-insufficient genes (HIGs) are involved in leukemogenesis is largely unknown and powerful experimental strategies aimed at their identification are currently lacking. Here, we present a new approach to discover HIGs, using retroviral integration mutagenesis in mice in which methylated viral integration sites and neighbouring genes were identified. In total we mapped 6 genes which are flanked by methylated viral integration sites (mVIS). Three of these, i.e., *Lrmp*, *Hcls1* and *Prkrir*, were up regulated and one, i.e., *Ptp4a3,* was down regulated in the affected tumor. Next, we investigated the role of *PTP4A3* in human AML and we show that *PTP4A3* expression is a negative prognostic indicator, independent of other prognostic parameters. In conclusion, our novel strategy has identified *PTP4A3* to potentially have a role in AML, on one hand as a candidate HIG contributing to leukemogenesis in mice and on the other hand as a prognostic indicator in human AML.

## Introduction

Acute myeloid leukemia (AML) is a complex disease driven by multiple cytogenetic abnormalities, such as inv(16), t(8;21), t(15;17), 3q abnormalities, deletions of (the q-arms) of chromosome 5 and 7 and by aberrant expression and/or mutations of genes e.g., *EVI1, FLT3, RAS, RUNX1, CKIT, WT1, CEBPA* and *NPM1* [1], [2]. The frequent occurrence of chromosomal deletions suggests that haplo-insufficiencies contribute to the pathogenesis of AML. However, because deleted regions often harbor numerous genes, it remains difficult to pin point critical haplo-insufficient genes (HIGs) involved in the pathogenesis of AML. Gene expression profiling (GEP) focusing on down regulated genes could be informative, however differences in expression levels may relate to differentiation status of the AML blasts, rather than to mechanisms underlying leukemogenesis [3]. In addition, mapping of minimal affected regions in combination with GEP to identify HIGs often is cumbersome because these regions may still contain numerous genes and differences in their expression level may be subtle. Even in chromosomal regions frequently lost upon leukemic progression, e.g., the q-arm of chromosome 7, identification of critical HIGs remains difficult.

Retroviral insertion mutagenesis in mouse models has been used to discover novel genes involved in the development of different types of cancer [4], [5], [6]. Most of these genes have been classified as proto-oncogenes, owing to the fact that proviral integrations preferentially occur in 5′ promoter regions, supposedly leading to increased or sustained expression of flanking genes. Only a small minority of identified genes have been classified as tumor suppressor genes or HIGs, based on disruption of coding sequences by the proviral integration [7], [8]. Gene therapy studies using murine leukemia virus (MLV)-based vectors have shown that epigenetic changes of long terminal repeats (LTRs) of integrated proviruses often result in silencing of therapeutic genes [9], [10], and that preventing methylation of the CpG islands within LTRs overcomes this problem [11]. Based on these observations, we hypothesized that methylation of viral sequences not only results in silencing of retroviral genes themselves but may also affect host genes located proximal to proviral integrations. Methylated LTRs located in proximity of promoter regions may thus identify genes that are deregulated leading to haplo-insufficiency.

To discover potential HIGs relevant for human AML, we used murine leukemia samples induced by Graffi 1.4 Murine Leukemia Virus (Gr1.4 MLV), classified as mixed lineage or myeloid leukemias by immunophenotyping [6], [12]. By methylation specific PCR (MSP) and methylated DNA immunoprecipitation (MeDIP) [13] we observed an extensive variation in the level of DNA methylated proviral integrations in these tumors. We designed a strategy to map methylated proviral integrations by combining MeDIP, inverse PCR (iPCR) and promoter array hybridization. We identified 6 genes to be flanked by methylated viral integration sites (mVIS), of which *Lrmp*, *Hcls1* and *Prkrir* were transcriptionally up regulated and *Ptp4a3* was transcriptionally down regulated. Further studies in human AML samples revealed a negative prognostic value of *PTP4A3* expression levels, independent of other prognostic indicators. In conclusion, by mapping DNA methylated viral integration sites in murine leukemias induced by retroviral integration mutagenesis followed by comparative analysis in human AML, we identified *PTP4A3* not only as a candidate HIG contributing to leukemogenesis in mice but also as an independent prognostic indicator in human AML.

## Results

### Viral integrations sites of the Graffi1.4 MuLV are subject to DNA methylation

**Figure 1:**
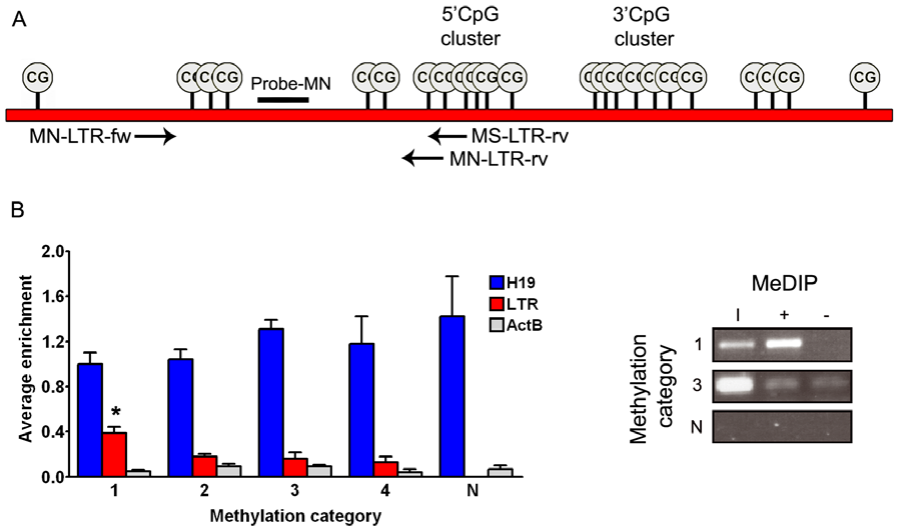
LTR methylation analysis. (A) Overview of the methylation specific PCR (MSP) approach. Depicted is a schematic representation of the Gr1.4 MuLV LTR, containing 23 CpGs. The MSP was performed, after bisulphite treatment, with a methylation neutral forward primer (MN-LTR-fw), and a methylation specific (MS-LTR-rv) or neutral (MN-LTR-rv) reverse primer. Amplification products were quantified using methylation neutral probe-MN. Tumor samples were divided into 4 equal groups based on the methylation status of their LTRs (1 = highest LTR methylation level, 4 = lowest LTR methylation level). (B) *Left panel.* For *H19*, the LTR and *ActB*, average enrichment after MeDIP compared to input levels were calculated for each MSP-defined methylation category as well as for normal bone marrow, spleen and liver (N). In category 1 to 4 respectively 18, 15, 4 and 3 samples were analysed; error bars indicate standard deviations. P-values were calculated using a Wilcoxon test; *significantly higher than other categories, p-value <0.001. *Right panel*. Example of LTR enrichment after MeDIP (I = input, + = IP with anti-5-methylcytidine, − = IP with pre-immune serum IgG, 1 and 3 = methylation categories, N = normal spleen).

In this study murine leukemia samples induced by Gr1.4 MLV were analysed [6]. First, a methylation specific PCR (MSP) was performed to determine the level of DNA methylation of the Gr1.4 MLV LTRs. To this end, amplification products from methylated LTRs were quantified with quantitative PCR (qPCR) and corrected for total LTRs in these samples (Figure 1A). A considerable variation in LTR methylation was seen between different tumors (data not shown). Based on these methylation levels, leukemia samples were divided into 4 methylation categories of equal sample size (1 = highest LTR methylation level, 4 = lowest LTR methylation level).

Subsequently, MeDIP was used on a subset of samples to enrich for methylated LTRs and flanking genomic regions. As a control, genomic DNA of normal bone marrow, spleen and liver was used. MeDIP enrichment relative to input levels was determined for the LTR, the non-methylated actin B locus (*ActB*) and the hemi-methylated imprinting control region 1 (ICR1) of *H19*. As expected, *H19* enrichment scores were high and *ActB* enrichment scores were low in all categories (Figure 1B). Additionally, samples in the highest methylation category showed a significantly higher LTR enrichment after MeDIP compared to the samples in other categories (p-value <0.001), confirming the specificity of the MSP (Figure 1B).

### *Ptp4a3* is flanked by a methylated viral integration site and is transcriptionally down regulated

**Figure 2:**
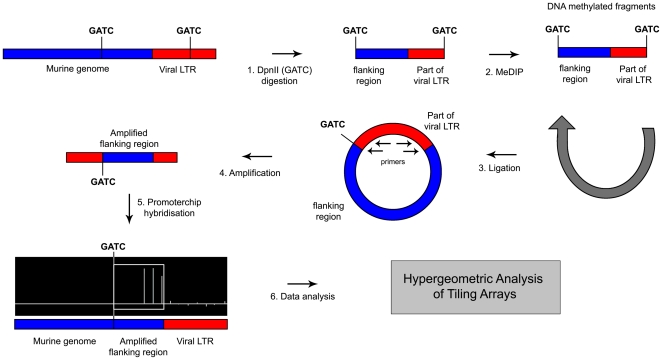
Identification of mVIS. Strategy outline for identification of regions flanking DNA methylated viral integration sites (mVIS) within murine leukemias. Genomic DNA was digested with DpnII (step 1), followed by methylated DNA immunoprecipitation (MeDIP, step 2). MeDIP enriched fragments were ligated (step 3) and amplified using primers within the LTR (step 4). These fragments were hybridized on a DNA promoter array (step 5). Hypergeometric Analysis of Tiling Arrays (HAT) was used to identify regions flanking mVIS (step 6).

**Figure 3:**
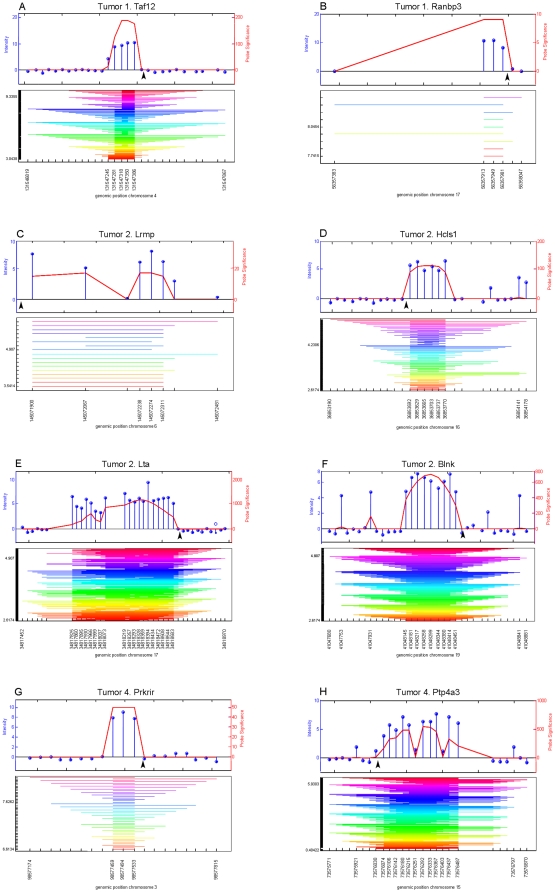
Identified viral integration sites. Eight viral integrations identified with HAT could be confirmed with directed PCR and Sanger sequencing (see Table S1 for further details). The graphical output of HAT is represented in graph A–H. Above each graph, the tumor in which the integration was identified as well as the nearby located gene are indicated. The upper panel of each graph shows normalized intensities of the different probes (blue lollipops) on the mouse promoter 1.0R arrays and their significance (in red) as calculated with HAT. The black arrowhead indicates the exact position of the proviral integration, as determined by directed PCR followed by Sanger sequencing. In the lower panel the lowest and highest probe intensity threshold with a significant outcome are given on the left. The stripes indicate significantly enriched regions at different probe intensity thresholds, calculated with HAT, which are merged into the final viral integration site. Below each graph, the genomic position is indicated (assembly mm8, February 2006).

Genes located near methylated viral integration sites (mVIS) may be down regulated due to the proximity of a methylated regulatory sequence, and, their transcriptional down regulation may contribute to murine leukemogenesis. Therefore, after showing that a proportion of viral integration sites are subject to DNA methylation, we set out to identify genes flanking these viral integration sites. To this end, iPCR, to amplify regions flanking viral integration sites, and MeDIP, to enrich for DNA methylated fragments, were combined to amplify regions flanking mVIS (Figure 2). Amplified fragments of 6 tumor samples were hybridized to Murine 1.0 R promoter arrays and, using hypergeometric analysis of tiling arrays (HAT) [14], 15 amplified regions were mapped in these tumors (Table S1). Eight of these integrations were validated by directed PCR followed by Sanger sequencing (Figure 3, Table S1). Because MLVs tend to integrate within 10 kb around the transcriptional start site [15], the nearest genes within 10 kb downstream of these 8 mVIS were determined (Figure 3, Table S1).

**Figure 4:**
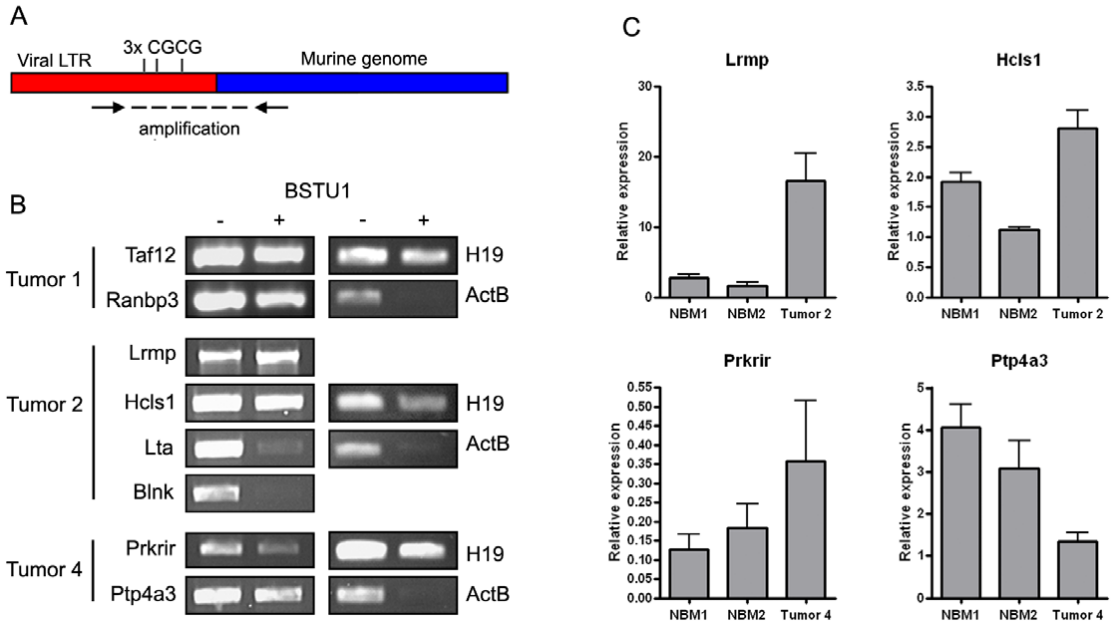
Methylation sensitive restriction analysis of viral integration sites and expression of nearby located genes. (A) Schematic overview of the methylation specific restriction approach. Genomic DNA was digested with BstU1 (CGCG, blocked by DNA methylation), followed by mVIS amplification with primers as indicated by arrows. If the flanking LTR is methylated, mVIS amplification is unaffected upon BstU1 digestion. (B) All 8 identified viral integration sites, identified in tumor 1, 2 and 4, were amplified before (−) and after (+) BstU1 digestion. As controls, *H19* (hemi-methylated) and *ActB* (unmethylated), both containing 2 BstU1 digestion sites, were analysed in each tumor. (C) Expression levels of 4 genes flanked by methylated viral integration sites were determined by qPCR in the respective tumors. Expression levels relative to housekeeping gene *Tbp* are shown; error bars indicate standard deviations. NBM = normal bone marrow.

To support that regions identified in this way were indeed flanked by methylated LTRs, we performed a methylation sensitive digestion followed by directed PCR. Using this approach, only viral integration sites flanked by methylated LTRs could be amplified (Figure 4A), as was the case for 6 out of 8 identified integrations (Figure 4B, Table S1). Subsequently, expression levels of genes flanking these mVIS were quantified by qPCR and compared to normal bone marrow expression levels. Unfortunately, RNA of tumor 1 was lacking, therefore this analysis could not be performed for *Taf12* and *Ranbp3.* Of the other 4 genes, *Ptp4a3* expression was 2–3 fold reduced in the respective tumor (Figure 4C, Table S1).

### *Ptp4a3* is an independent prognostic factor in human AML

**Figure 5:**
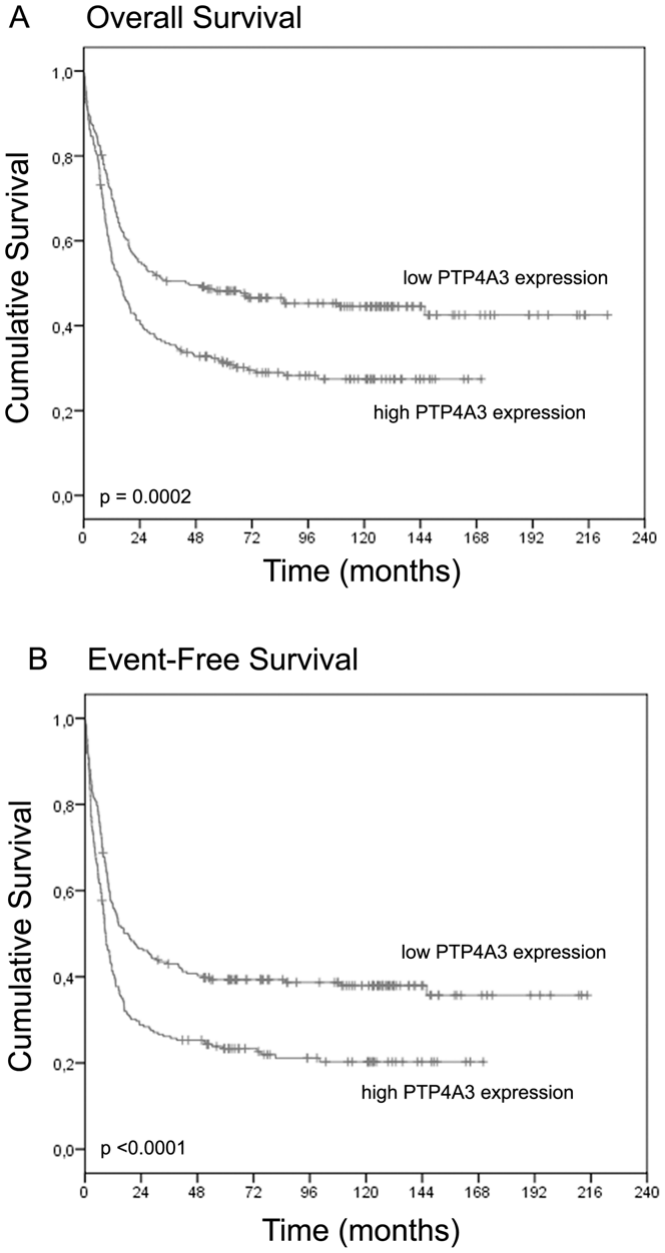
Survival analysis. A cohort of 454 de novo AML cases diagnosed under the age of 60 was divided into 2 groups of equal size based on MAS5 normalised expression of *PTP4A3* (probe 206574_s_at). Overall survival (A) and event-free survival (B) were analysed. P-values were calculated with a log rank test.

The human orthologue of murine *Ptp4a3*, i.e., *PTP4A3*, was further studied in human AML. Transcript levels of *PTP4A3* were assessed in 454 AML samples, diagnosed under the age of 60, profiled using the HGU133 2.0 plus gene expression arrays [16]. *PTP4A3* expression values are represented by 2 probesets with a high correlation (Pearson correlation coefficient = 0.90). Survival analysis with these probesets gave similar results; all results shown are based on expression levels of probeset 206574_s_at. *PTP4A3* expression levels were negatively correlated with prognostic outcome both for overall survival (OS, p-value <0.0001, hazard ratio = 1.269) and event-free survival (EFS, p-value <0.0001, hazard ratio = 1.261). Kaplan-Meier curves are shown in Figure 5. A permutation test predicted a probability of 0.0036 for a random gene locus to be a significant prognostic indicator with a p-value <0.0001 for both OS and EFS. Multivariate analysis showed that the negative correlation of *PTP4A3* expression with event-free survival was independent of other prognostic parameters, i.e., age, white blood cell count, cytogenetic risk, *CEBPA* mutation status and *NPM1^+^FLT3ITD^−^* status (Table 1).

**Table 1:** Multivariate survival analysis.

	Overall Survival		Event-Free Survival	
Risk factor	HR (95% CI)	P-value	HR (95% CI)	P-value
*PTP4A3* expression	1.112 (0.995–1.243)	0.061	1.131 (1.019–1.255)	0.021*
Age (decades)	1.134 (1.024–1.256)	0.016*	1.068 (0.969–1.177)	0.186
WBC∞	1.373 (1.063–1.773)	0.015*	1.296 (1.020–1.648)	0.034*
Favorable cytogenetic risk†	0.376 (0.257–0.548)	<0.0001*	0.469 (0.335–0.658)	<0.0001*
Unfavorable cytogenetic risk†	1.432 (1.059–1.935)	0.020*	1.507 (1.124–2.020)	0.006*
*NPM1*+*FLT3ITD*-‡	0.473 (0.317–0.705)	0.0002*	0.578 (0.398–0.839)	0.004*
*CEBPA* double mutant$	0.591 (0.418–0.836)	0.003*	0.560 (0.384–0.815)	0.002*

Multivariate analysis in 454 de novo AML patients under the age of 60.

∞ WBC higher than 20×10^9^/L versus lower than 20×10^9^/L,

† compared to intermediate cytogenetic risk,

‡ compared to no *NPM1* ^+^ *FLT3ITD* ^−^,

$ compared to no *CEBPA* double mutation.

*Statistically significant. HR = hazard ratio, CI = confidence interval, WBC = white blood cell count, *FLT3ITD* = internal tandem duplication of *FLT3*.

## Discussion

We designed a strategy to identify candidate HIGs in AML using retroviral integration mutagenesis, by mapping DNA methylated proviral integrations. By using HAT [14], we deliberately aimed at detecting integrations present in the majority of the leukemic cells, which are most likely involved in the early phase of leukemogenesis. At the same time, integrations present in subclones that contribute to later stages of leukemic progression will be missed using this approach. We identified 6 genes that are flanked by methylated viral integrations. Expression analysis showed that *Lrmp* (lymphoid-restricted membrane protein), *Hcls1* (hematopoietic cell specific Lyn substrate 1) and *Prkrir* (protein-kinase, interferon-inducible double stranded RNA dependent inhibitor, repressor of (P58 repressor)) were up regulated and *Ptp4a3* (protein tyrosine phosphatase type IVA), a phosphatase also known as *Prl3* (phosphatase of regenerating liver 3) was down regulated in the respective murine tumor. These results indicate that a flanking methylated viral integration site does not necessarily lead to transcriptional repression. As 1 out of 4 genes flanked by a mVIS was transcriptionally down regulated and expression of the 2 other genes could not be investigated, the efficiency to detect potential HIGs by identifying mVIS would approximately be 17–25%. However, the number of analysed tumors is too small to allow an accurate estimation of the efficiency.

*Ptp4a3* expression is controlled by p53 induced after DNA damage in mouse embryonic fibroblasts (MEFs) and its activity is involved in inducing a G1 cell cycle arrest in these cells [17]. Surprisingly however, the same study also demonstrated a cell cycle arrest upon reduction of *PTP4A3* expression [17]. Apparently, depending on expression level dosage, *PTP4A3* may have both positive and negative effects on cell cycle regulation. Hence, *PTP4A3* haplo-insufficiency, but not its complete loss, may lead to an impairment of cell cycle arrest after DNA damage. Dosage effects of *PTP4A3* expression in relation to cellular responses may be more complex, particularly in cancer cells. For example, in carcinoma cell lines *PTP4A3* expression may lead to down regulation of p53 [18] and it is variably induced by γ-irradiation [19]. Finally, high *PTP4A3* expression has been linked to increased tumor aggressiveness in different types of solid tumors, e.g., melanoma, gastric cancer, colon cancer, hepatocellular carcinoma and breast cancer [20], [21], [22], [23], [24], possibly because high *PTP4A3* expression leads to increased epithelial-mesenchymal transition [25].

The role of *PTP4A3* in hematopoietic malignancies has not been studied as extensively as in carcinoma. Only a few studies report differences in expression levels of *PTP4A3* in ALL and myeloma subgroups, based on gene expression profiling [26], [27], [28]. Interestingly however, in a recent study, *PTP4A3* has been proposed to have a role in drug-resistance in AMLs with internal tandem duplication of *FLT3* (*FLT3ITD*) [29]. This finding, together with the observation that high *PTP4A3* expression negatively correlates with prognostic outcome, indicates that PTP4A3 might be a potential therapeutic target in AML.

In conclusion, using a retroviral mutagenesis screen in which we enriched for DNA methylated viral integration sites we identified *PTP4A3* as a potential haplo-insufficient gene with an independent prognostic value in human de novo AML. Challenges for the future are to determine the dose-effect of *PTP4A3* expression in myeloid development and to extend the screens to additional myeloid neoplasms, e.g., myelodysplasia, therapy-related AML, AML secondary to bone marrow failure and myeloproliferative disorders.

## Materials and Methods

### Ethics statement

For this study no novel murine leukemias were generated, all experiments described were performed on material generated in a previous study [6]. All animal procedures for the use of control bone marrow fractions were approved by the animal care and use committee of the Erasmus MC (approval # 119-10-05).

All human cell samples were obtained after written informed consent and stored anonymously in a biobank. The study was performed under the permission of the Institutional Review Board of the Erasmus MC, registration number MEC-2008-387.

### Mouse leukemia and normal cell samples

DNA and RNA samples from a previously generated panel of Gr1.4-induced leukemia's [6], and control samples (bone marrow, spleen, liver) from normal FVB/N mice were used.

### Methylation specific PCR

Primer and probe sequences are shown in Table S2. Two µg of genomic DNA was treated with bisulphite using the EZ DNA Methylation kit according to the manufacturer's protocol (Zymo research, Orange, CA, USA). LTRs were amplified with bsLTRfw and bsLTRrv using 1 µL out of 10 µL of bisulphite-treated DNA. Cycling conditions were 30″ at 94°C, 30″ at 50°C and 1′ at 72°C for 10 cycles in a total volume of 50 µL. Two µL was used in a nested qPCR (Figure 1A) using MN-LTR-fw×MS-LTR-rv/MN-LTR-rv (MN = methylation neutral, MS = methylation specific). Cycling conditions were 15″ at 94°C, 30″ at 57°C and 30″ at 60°C for 45 cycles. Amplified LTRs, methylated and unmethylated, were quantified using a methylation neutral probe (probe-MN, Sigma-Aldrich, Zwijndrecht, The Netherlands). Delta cycle threshold-values (dCt), representing the number of methylated LTRs as a fraction of total LTRs, were calculated as follows: dCt = Ct(Methylated LTRs)-Ct(All LTRs) = Ct(MN-LTR-fw×MS-LTR-rv) – Ct(MN-LTR-fw×MN-LTR-rv). PCRs were performed in duplicate and mean dCt values were calculated.

### MeDIP

Ten µg genomic DNA was digested overnight with 100 U of DpnII (New England Biolabs, Ipswich, MA, USA). Four µg digested DNA was denatured for 10′ at 95°C and incubated with either 2.5 µg anti-5-methylcytidine (BI-MECY-1000, Eurogentec, Liège, Belgium) or mouse pre-immune IgG (Sigma-Aldrich, Zwijndrecht, The Netherlands) in 500 µL IP-buffer (PBS with 0.05% Triton X-100) for 2 hrs at 4°C, followed by incubation with 30 µL of washed beads (M-280 sheep-anti-mouse IgG, Invitrogen, San Diego, CA, USA) for 2 hrs at 4°C. Beads were washed 3 times with 700 µL IP-buffer. As a 10% input reference, 400 ng digested DNA not subjected to MeDIP was used. Beads and the 10% input reference DNA were resuspended in 100 µL IP-buffer and incubated for 3 hrs at 50°C after adding 20 µg proteinase K (Roche, Basel, Switzerland). Supernatants, containing immunoprecipitated DNA, and the input DNA were purified using the MinElute Reaction Cleanup Kit (Qiagen, Hilden, Germany) and were eluted in 40 µL elution buffer. Two µL immunoprecipitated DNA was used to amplify the imprinting control region 1 (ICR1) of *H19* with *H19*ICR1fw × *H19*ICR1rv, *ActB* with *ActB*fw × *ActB*rv and the LTR with LTRfw × LTRrv using (q)PCR. Primer sequences are shown in Table S2. Cycling conditions were 30″ at 95°C, 30″ at 58°C and 45″ at 72°C for 30 cycles (PCR) or 15″ at 94°C, 30″ at 59°C and 30″ at 60°C for 45 cycles (qPCR). Amplification products were analysed using gel electrophoresis (PCR) or quantified (qPCR) using SYBRgreen Master mix (Applied Biosystems, Foster City, CA, USA).

### Inverse PCR

Primer sequences are shown in Table S2. Six murine leukemias with high LTR enrichment (more than 10% of input) and low *ActB* enrichment (less than 10% of input) were selected for inverse PCR. Eight µL MeDIP-DNA was denatured for 3′ at 95°C, renatured by a temperature decrease of 0.1°C/sec to 20°C, and ligated for 45′ at room temperature using a rapid DNA ligation kit (Roche, Basel, Schwitzerland). Two µL out of 20 µl ligated product was amplified with primers mL1 and mL2, followed by a nested PCR with primers mL1N and mL2N using 2 µL of the first PCR product. Cycling conditions were 30″ at 95°C, 30″ at 60°C (first PCR) or 56°C (nested PCR) and 3′ at 72°C for 30 cycles. In the nested PCR 10 mM dCTP, dATP, dGTP, 8 mM dTTP and 2 mM dUTPs were used.

### Promoter array hybridization

PCR products of 10 nested PCR reactions were purified with a PCR purification kit (Qiagen, Hilden, Germany) and pooled. A total of 7.5 µg of these amplified fragments was fragmented and labeled using the GeneChip WT Double-stranded DNA terminal labeling kit (Affymetrix, Santa Clara, CA, USA). Fragmentation to 66 bp was checked on a Bioanalyser (Agilent, Santa Clara, CA). Labeled DNA was hybridized to mouse promoter 1.0R arrays (Affymetrix, Santa Clara, CA, USA) for 16 hrs at 45°C. Arrays were washed with the FS_450_0001 protocol using the Fluidics Station 450 (Affymetrix, Santa Clara, CA, USA), followed by scanning. Probe values were normalized with model-based analysis of tiling-arrays (MAT) [30] and mVIS were determined using hypergeometric analysis of tiling arrays (HAT) [14], both for HAT and MAT default settings were used. Genes located nearby amplified regions were identified using UCSC (assembly mm8, Feb. 2006).

### Directed PCR and Sanger sequencing

Primers are shown in Table S2; amplification of the integration site was performed with VIS(*corresponding gene*) × LTRfw2, for *Lrmp* a nested PCR was performed with VIS(*Lrmp_nested*) × LTRfw. As input, 200 ng of the corresponding tumor DNA was used; cycling conditions were 30″ at 95°C, 30″ at 58°C and 45″ at 72°C for 30 cycles. Products were purified using the Multiscreen HTS 66-well filtration system (Millipore, Billerica, MA, USA). Sanger sequencing was performed with primer LTRfw according to the manufacturer's protocol (Applied Biosystems, Foster City, CA, USA).

### Methylation sensitive restriction analysis

Primers are shown in Table S2. Two and a half µg of tumor DNA was digested with 25 U of BstU1 (New England Biolabs, Ipswich, MA, USA) o/n at 60°C, purified using the Multiscreen HTS 66-well filtration system (Millipore, Billerica, MA, USA), eluted in 30 µl and diluted to 50 ng/µl. Amplification of the integration site was performed as described under directed PCR and Sanger sequencing, with 100 instead of 200 ng input of DNA. As controls *H19* ICR1 (*H19*ICR1fw × *H19*ICR1rv) and *ActB* (*ActB*fw × *ActB*rv) were amplified. Cycling conditions were 30″ at 95°C, 30″ at 58°C and 45″ at 72°C for 30 cycles. Amplification products were analysed using gel electrophoresis.

### RNA isolation, cDNA preparation and qPCR

RNA of murine samples was isolated using Trizol (Invitrogen, San Diego, CA) according to the manufacturer's protocol. One µg of RNA was used for cDNA preparation, using SuperScript II Reverse Transcriptase (Invitrogen, San Diego, CA) according to the manufacturer's protocol. One µl cDNA was used as input for the qPCR. Genes of interest were amplified with their respective forward and reverse primers (Table S2), as an input control, TATA box binding protein (*Tbp*) was analysed. Cycling conditions were 3″ at 95°C and 30″ at 60°C for 45 cycles. Amplification products were quantified using Fast SYBRgreen Master mix (Applied Biosystems, Foster City, CA, USA). Expression levels relative to *Tbp* were calculated.

### Survival analysis human AML samples

Purified AML blasts were obtained following informed consent as described [31]. Gene-expression profiles of 454 de novo AML patients under the age of 60 were used for this analysis [16]. Expression levels were MAS5 normalised (Scaling factor 100), values <30 were set at 30, followed by log2 transformation.

For *Ptp4a3*, univariate and multivariate survival analyses were performed using expression levels of probesets 206574_s_at or 209695_at in a Cox regression model. In the multivariate analysis age, white blood cell count, cytogenetic risk group, *NPM1* ^+^ *FLT3ITD^−^* status and *CEBPA* mutation status were used as additional prognostic parameters. We recognised the following cytogenetic risk groups: favorable = t(15;17), inv(16) and t(8;21), unfavorable = t(3;3), inv(3), −7/7q-, −5/5q-, complex karyotype, t(11q23) except t(9;11), t(9;22) and t(6;9), intermediate = all other cases with known cytogenetics. Kaplan-meier graphs were generated by dividing the AML cohort in 2 groups of equal sample size based on *PTP4A*3 expression of probe 206574_s_at. Analyses were performed in SPSS (version 17, SPPS Inc, Chicago, IL).

For the permutation test, all probesets with an annotated gene symbol (based on HG-U133_Plus_2.na32.annot.csv, Affymetrix, Santa Clara, CA, USA) were selected. Next probesets with expression levels <30 in all 454 patients were discarded, leaving a total of 40720 probesets. The permutation test was performed by randomly selecting 6 probesets (representing 6 mVIS), followed by randomly selecting 1 out of these 6 probesets (representing 1 down regulated gene). For this probeset a univariate Cox regression analysis was performed for overall survival (OS) and event-free survival (EFS). A p-value of <0.0001 (as observed for *PTP4A3*) was considered significant. This analysis was repeated 100.000 times, followed by calculating the frequency, i.e., probability, of observing a significant p-value for both OS and EFS. Analyses were performed in Matlab (version 2008b, Mathworks, Natick, MA).

## Supporting Information

Table S1 **Retroviral integrations.** Retroviral integrations identified with HAT are listed. For each integration the murine tumor and the genomic position are indicated as well as whether the integration could be confirmed with directed PCR and Sanger sequencing. For all integrations that could be confirmed, nearby located genes are given, their distance to the retroviral integration and whether the flanking viral integration was DNA methylated as analysed by methylation sensitive restriction analysis. Finally, for the 6 genes with a flanking DNA methylated viral integration site is indicated if they were down regulated in the respective tumor.
(XLS)

Table S2 **Primers and probes.**
(XLS)
